# *Mycobacterium tuberculosis* Infection Up-Regulates Sialyl Lewis X Expression in the Lung Epithelium

**DOI:** 10.3390/microorganisms9010099

**Published:** 2021-01-04

**Authors:** Rita Matos, Kaori L. Fonseca, Stefan Mereiter, Ana Raquel Maceiras, Joana Gomes, Cristina Vilaplana, Fátima Gartner, Pedro N. S. Rodrigues, Celso A. Reis, Margarida Saraiva, Ana Magalhães

**Affiliations:** 1 i3S—Instituto de Investigação e Inovação em Saúde, Universidade do Porto, 4200-135 Porto, Portugal; ritam@ipatimup.pt (R.M.); kaorifonseca@gmail.com (K.L.F.); stefan.mereiter@imba.oeaw.ac.at (S.M.); raquel.maceiras@gmail.com (A.R.M.); joanag@ipatimup.pt (J.G.); fgartner@ipatimup.pt (F.G.); prodrigu@ibmc.up.pt (P.N.S.R.); celsor@ipatimup.pt (C.A.R.); 2 IPATIMUP—Instituto de Patologia e Imunologia Molecular da Universidade do Porto, 4200-135 Porto, Portugal; 3 ICBAS—Instituto de Ciências Biomédicas Abel Salazar, University of Porto, 4050-313 Porto, Portugal; 4 IBMC—Instituto de Biologia Molecular e Celular, Universidade do Porto, 4200-135 Porto, Portugal; 5 Programa de Pós-Graduação Ciência para o Desenvolvimento (PGCD), Instituto Gulbenkian de Ciência (IGC), 2780-156 Oeiras, Portugal; 6 IGTP—Experimental Tuberculosis Unit, Universitat Autònoma de Barcelona, CIBER Enfermedades Respiratorias, Fundació Institut d’Investigació en Ciències de la Salut Germans Trias i Pujol, 08916 Barcelona, Spain; cvilaplana@igtp.cat; 7 FMUP—Faculdade de Medicina da Universidade do Porto, 4200-319 Porto, Portugal

**Keywords:** *Mycobacterium tuberculosis*, lung glycophenotype, Lewis antigens, Sialyl-Lewis X

## Abstract

Glycans display increasingly recognized roles in pathological contexts, however, their impact in the host-pathogen interplay in many infectious diseases remains largely unknown. This is the case for tuberculosis (TB), one of the ten most fatal diseases worldwide, caused by infection of the bacteria *Mycobacterium tuberculosis*. We have recently reported that perturbing the core-2 *O*-glycans biosynthetic pathway increases the host susceptibility to *M. tuberculosis* infection, by disrupting the neutrophil homeostasis and enhancing lung pathology. In the present study, we show an increased expression of the sialylated glycan structure Sialyl-Lewis X (SLeX) in the lung epithelium upon *M. tuberculosis* infection. This increase in SLeX glycan epitope is accompanied by an altered lung tissue transcriptomic signature, with up-regulation of genes codifying enzymes that are involved in the SLeX core-2 *O*-glycans biosynthetic pathway. This study provides novel insights into previously unappreciated molecular mechanisms involving glycosylation, which modulate the host response to *M. tuberculosis* infection, possibly contributing to shape TB disease outcome.

## 1. Introduction

Glycosylation is a common post-translation modification, recognized to regulate several key biological processes, either on homeostasis or pathological conditions [1,2,3]. This highly regulated enzyme-mediated modification occurs mainly on the Golgi apparatus and results in the production of glycosidic linkages of saccharides to other saccharides, proteins or lipids [4]. Glycans and glycoconjugates can be classified into several classes, including the protein linked *N*-glycans and *O*-glycans. The biosynthesis and modification of glycan structures are tissue- and cell-specific and are dictated by the cellular glycosylation machinery [5].

The crosstalk between glycosylation, infection, inflammation and immune surveillance is well established [6,7,8,9]. Glycans are important mediators in host pathogen interactions, constituting ligands that are explored by the pathogen for the colonization of host tissues, as well as important effector molecules for cellular signaling in response to infection [10,11]. Moreover, glycans are key players in immune cell adhesion and recruitment to the site of infection. We have previously described a critical role of glycan structures in the context of two infections with high relevance to human health: *Helicobacter pylori* and *Mycobacterium tuberculosis*. In the case of *H. pylori*, we showed that infection results in increased expression of sialylated Lewis antigens in the gastric epithelium, which are recognized by the bacterial sialic acid binding adhesin (SabA), contributing to a close contact with the gastric mucosal cells and a tighter fit of the infection load with proficient transfer of virulence factors [12,13]. As for *M. tuberculosis*, a pathogen that causes over 1.4 million deaths and over 10 million new cases of tuberculosis (TB) per year [14], we have recently described a link between *O*-glycans and susceptibility to infection [15]. Briefly, the deficiency of *Gcnt1*, a key enzyme acting on the initial biosynthetic steps of the core-2 *O*-glycans, was associated with increased susceptibility to *M. tuberculosis* infection, characterized by augmented lung immune pathology, with both hematopoietic and non-hematopoietic compartments being crucial for this process [15]. Previous studies had shown that a combined deficiency in fucosyltransferases 4 and 7 in the mouse model led to accelerated death following *M. tuberculosis* infection, which was not caused by increased bacterial proliferation, nor by exacerbated tissue pathology [16]. This combined deficiency was subsequently shown to be associated with reduced numbers of T cells and antigen-specific effector responses in lymph nodes, but normal T cell responses in the lung [17]. Interestingly, all these glycosyltransferases (Gcnt1, Fut4 and Fut7) are involved in the biosynthesis of Lewis antigens [18]. Moreover, it has been recently shown that *Mycobacterium bovis* infection led to increased expression of specific Lewis epitopes on *N*-glycans [19].

Lewis antigens are terminal fucosylated structures that decorate glycan chains, and their expression has also been implicated in the recognition and binding of several pathogens [11,12]. These antigens are classified based on their biosynthesis: Type 1 chains are characterized by the Galβ1,3GlcNAc linkage, while type 2 chains display a Galβ1,4GlcNAc linkage [3,20]. The addition of a fucose to terminal galactose (Gal) on type 1 Galβ1-3GlcNAc chain leads to H-type 1 structure, that can be further modified with a fucose on the *N*-acetylglucosamine (GlcNAc) residue resulting on the di-fucosylated Lewis B (LeB) antigen. Alternatively, an α1,4-fucosyltransferase can act towards the type 1 chain and add a fucose to the GlcNAc residue and lead to the formation of Lewis A (LeA) antigen. The modification of a type 1 chain by an alpha2,3-sialyltransferase followed by addition of fucose results on the biosynthesis of sialyl-Lewis A (SLeA). The biosynthesis of the type 2 Lewis antigens is very similar, with addition of the same glycan units, but it occurs in the type 2 Galβ1-4GlcNAc chains, originating the isomers Lewis X (LeX), Lewis Y (LeY) and sialyl-Lewis X (SLeX) [20,21]. Among the Lewis antigens, SLeX is well studied in inflammation and in infection. Indeed, SLeX mediates the interactions between host cells and some pathogens like *H. pylori* [12,22].

Despite the growing body of evidence attesting a role of terminal sialylated Lewis antigens in infection, the crosstalk between *M. tuberculosis* and the lung sialylation profile has never been assessed. Here, we investigated how *M. tuberculosis* infection impacted the expression of different glycan structures in the lung, using a mouse model of infection. We demonstrate that *M. tuberculosis* infection promotes the expression of the sialylated glycan structure SLeX, but not of the other Lewis antigens. This increase in the SLeX glycan epitope was observed in infection by two different *M. tuberculosis* strains, H37Rv and HN878, and in two distinct genetic mouse backgrounds with different infection susceptibility, C57BL/6 and C3HeB/FeJ. Furthermore, we showed that the up-regulation of SLeX tissue expression is concomitant with the higher expression levels of enzymes involved in the biosynthetic pathway of SLeX on core-2 *O*-glycans. Noteworthy, this increase in the lung epithelium sialylation was also observed in mice lacking Gcnt1 glycosyltransferase, suggesting that the overall increased lung sialylation in response to *M. tuberculosis* infection results from up-regulation of SLeX terminal decoration on different glycoconjugate carriers. This work further illustrates the existing cross-talk between glycans and infection, highlighting the importance of glycosylation in infectious diseases.

## 2. Materials and Methods

### 2.1. Ethics Statement

All animal experiments were performed in accordance with recommendations of the European Union Directive 2010/63/EU, and were approved by the i3S Animal Ethics Committee and the Portuguese National Authority for Animal Health (# 014811/2016-07-13) or by the Animal Experimentation Ethics Committee of the Hospital Universitari Germans Trias i Pujol (#B9900005) and the Dept d’Agricultura, Ramaderia, Pesca, Alimentació i Medi Natural of the Catalan Government.

### 2.2. Animals

C57BL/6 wild-type and *Gcnt1^-/-^* mouse strains were bred and housed at the i3S animal house and infected under ABSL3 conditions. The *Gcnt1^-/-^* mouse [23] was obtained from the Consortium for Functional Glycomics (CFG). C3HeB/FeJ specific-pathogen-free mice (6–8 weeks old) were obtained from Jackson Laboratories (Bar Harbor, ME, USA). Mice were supervised daily following a strict monitoring protocol in order to ensure animal welfare and euthanized with isoflurane (inhalation excess) or by CO_2_ inhalation. Food and water were ad libitum.

### 2.3. Bacteria and Bacterial Growth

*M. tuberculosis* H37Rv and HN878 were grown in Middlebrook 7H9 liquid media (Becton-Dickinson, Sparks, MD, USA) supplemented with 0.05% Tween 80, 0.2% glycerol and 10% oleic acid/albumin/dextrose/catalase (OADC) enrichment for 7–10 days and then sub cultured in Proskauer Beck (PB) medium, supplemented with 0.05% Tween 80 and 2% glycerol, to the mid-log phase. To determine the concentration of *M. tuberculosis*, 6 frozen aliquots were serial diluted and plated in Middlebrook 7H11 (Becton-Dickinson, Sparks, MD, USA) agar plates supplemented with 10% OADC and 0.5% glycerol. Viable bacteria were determined by CFU enumeration after 21–28 days of incubation at 37 °C.

### 2.4. M. tuberculosis Infection

Mice were infected with *M. tuberculosis* H37Rv or HN878 strains via aerosol route using an inhalation exposure system (Glas-Col), following previously published protocols [15]. A murine model of active TB mimicking human TB was used, as described in Kroesen et al. [24]. C3HeB/FeJ mice were challenged with a single i.v. infection (4 × 10^4^–2 × 10^5^ CFU/mL per mouse) with *M. tuberculosis* H37Rv Pasteur strain. At the experimental time points (15, 30, 60 and 90 days post-infection), the lungs of infected animals were harvested and the bacterial load determined by CFU enumeration, as previously described [15,25,26]. The data is presented in Supplementary Figure S1.

### 2.5. Tissue Samples and Immunohistochemistry

At the indicated time-points, the lungs of control or infected animals were aseptically excised, fixed in 10% buffered-formalin and paraffin-embedded. Serial consecutive sections of 3 µm-thickness were made and used for immunohistochemical analysis. Lung sections were deparaffinized, rehydrated and the endogenous peroxidase activity was blocked with 3% H_2_O_2_ in methanol for 30 min. Then, sections were incubated with normal rabbit serum diluted 1:5 in BSA 10%-PBS for 30 min followed by incubation with primary antibody (see Table 1) diluted in BSA 5%-PBS overnight at 4 °C. Sections were incubated with secondary antibody rabbit anti-mouse (DAKO, Denmark) diluted 1:200 in BSA 5%-PBS at room temperature followed by avidin/biotin complex detection (Vector laboratories, Vectastain, CA, USA). Color reaction was developed for 10 min with 3,3-diamino-benzidine chromogen (DAB, Sigma, St. Louis, MO, USA) activated with 0.02% of H_2_O_2_ and counter staining of the nucleus was performed using Mayer’s Haematoxylin, followed by samples dehydration and mounting, as previously described [27].

The intensity of the immunohistochemistry staining was measured using ImageJ image analysis software with the Fiji image-processing package [28]. First, the area of interest was selected for each image and all brown tones were extracted by defining following cut-offs: Hue 1 to 45 and 205 to 255; brightness 0 to 122. Next, the brown saturation was divided in 11 categories, from lowest to highest saturation, and the number of pixels within each category was quantified. This gave rise to an intensity histogram, which was used to calculate the mean brown intensity value for the area of interest of each image. Three images of each animal per time-point were used, and at least five animals of each group were analyzed.

### 2.6. RNA Extraction, cDNA Synthesis and Real-Time PCR Analysis

At the indicated time-points, the lungs were aseptically excised and cell suspensions prepared as before [25,26]. Total RNA was extracted from lung of C57BL/6 and *Gcnt1^-/-^* mouse models, using TRI Reagent (Sigma, St. Louis, MO, USA) and converted to cDNA using the ProtoScript First Strand cDNA Synthesis Kit (E6300S, New England Biolabs, Ipswich, MA, USA). For real-time PCR analysis cDNA samples were amplified with PowerUp SYBRGreen Master Mix (Applied Biosystems, Foster City, CA, USA), using an ABI Prism 7500 Sequence Detection System (Applied Biosystems, CA, USA). Oligonucleotides were selected based on Nairn et al. [18], and their sequences are listed in Table 2. Specificity of amplification was confirmed by melting curve analysis. Standard curves and RQ value were determined for each gene. Ubiquitin was used as a reference gene for normalization of target gene abundance.

### 2.7. Statistical Analysis

Data were analyzed using GraphPad Prism 7. Differences between groups were analyzed with One-way ANOVA using Mann-Whitney test for multiple comparisons. Differences were considered significant for *p* ≤ 0.05 and represented as follows: * *p* ≤ 0.05; ** *p* ≤ 0.01; *** *p* ≤ 0.001 and **** *p* ≤ 0.0001.

## 3. Results

### 3.1. M. tuberculosis Infected Lungs Display Increased Sialylation

Lewis antigens are terminally fucosylated carbohydrate structures that decorate glycan chains on lipids or proteins. Although Lewis antigens have been associated with the protective roles of epithelial layers and secreted mucus, they are also recognized as important mediators of pathogen binding and the initiation of infection [31]. Although the expression of these glycan structures was shown to be affected in the context of disease, as in cancer and some infections [3,12], whether terminal Lewis antigens are modulated in TB remains largely unknown. To start unveiling this issue, we assessed using immunohistochemistry the presence of Lewis antigens in the lungs of C57BL/6 mice uninfected or infected via aerosol with two different strains of *M. tuberculosis* (H37Rv and HN878).

**Figure 1:**
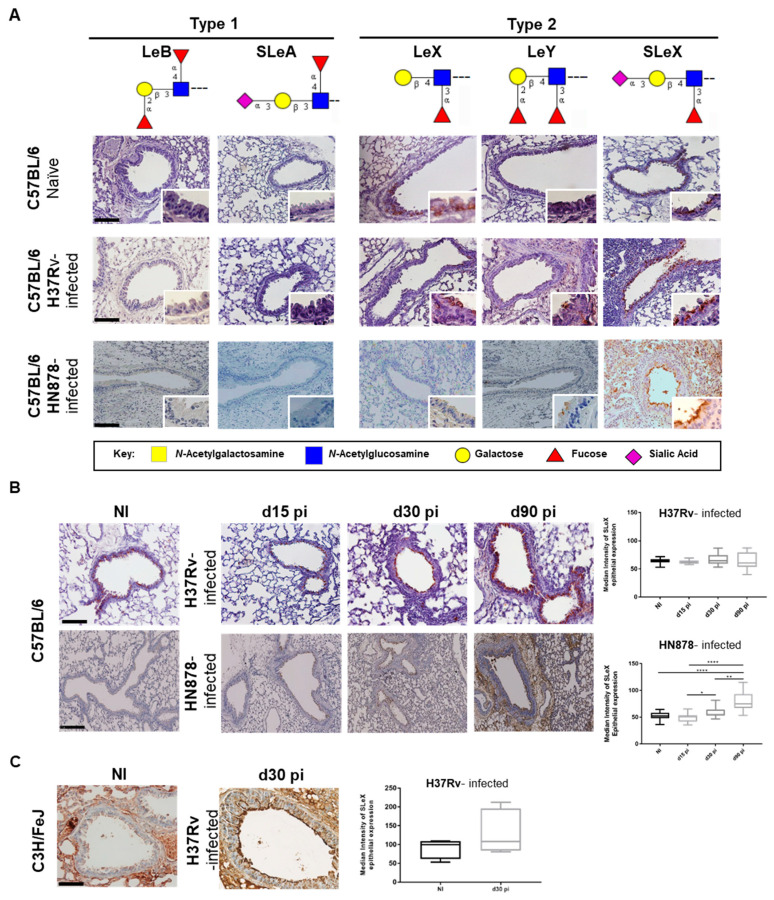
*M. tuberculosis* infection upregulates Silayl-Lewis X (SLeX) expression in lung tissue. (**A**) Lungs of C57BL/6 mice non-infected or infected after 90 days with two different *M. tuberculosis* strains (H37Rv or HN878) were stained by immunohistochemistry, using a panel of mAbs recognizing type 1 (Lewis B (LeB) and Sialyl-Lewis A (SLeA)) and type 2 (Lewis X (LeX), Lewis Y (LeY) and Sialyl-Lewis X (SLeX)) Lewis antigens. Magnification 200× and inserts at 400×. (**B**) SLeX expression on lung epithelium of non-infected or infected C57BL/6 and C3HeB/FeJ mice. C57BL/6 animals were infected via aerosol with *M. tuberculosis* H37Rv or HN878 strains. Lungs were recovered at the indicated time-points, fixed and lung sections stained for SLeX. Magnification 200×. The right panel represents the quantification of epithelial expression of SLeX. (**C**) C3HeB/FeJ mice were infected intravenously with *M. tuberculosis* H37Rv strain. Lungs were recovered at the indicated time-points, fixed and lung sections stained for SLeX. Magnification 200×. The panel on the right represents the quantification of epithelial expression of SLeX. Each dot represents the median intensity obtained per photograph. At least 5 mice were analyzed. Statistical analysis was performed using one-way ANOVA with Mann-Whitney test for multiple comparisons. *, *p* < 0.05; **, *p* < 0.01; ****, *p* < 0.0001. Monosaccharide symbols follow the Symbol Nomenclature for Glycans [38].

Type 1 Lewis antigens (Lewis B and Sialyl-Lewis A) were not detected in the lungs of non-infected mice, whereas the type 2 Lewis antigens (Lewis X, Lewis Y and SLeX) were present in the apical side of epithelial cells of bronchi and bronchiole (Figure 1A). In particular, SLeX was the most expressed Lewis antigen, with strong apical labelling of most surface epithelial cells and secreted mucus (Figure 1A). To investigate whether *M. tuberculosis* infection impacted the expression of these terminal structures, we evaluated their expression in lung sections of infected mice. As depicted in Figure 1A, 90 days post-infection there were no visible differences on the expression of the various Lewis antigens, except for SLeX which intensity on positive cells was augmented, particularly evident upon infection with *M. tuberculosis* HN878 strain.

Thus, we further addressed the dynamics of SLeX expression during the course of infection. This evaluation was performed using the CSLEX-1 antibody. Although we and others have shown that SLeX is expressed in human neutrophils [15], using the CSLEX-1 antibody we could not assess such an expression in the mouse model. This is explained by the fact that the majority of SLeX in mice neutrophils displays an *O*-acetylated form of sialic acid, which is not recognized by the CSLEX-1 antibody [32,33,34]. Thus, our analysis was focused on the lung epithelium. We observed an increased expression of SLeX antigen over time in the lung epithelia of C57BL/6 mice infected with *M. tuberculosis* (Figure 1B). Interestingly, this increase was much more evident and significant in the case of infection with the *M. tuberculosis* strain HN878. In particular, automatic quantification of SLeX staining, showed a significantly increased expression of this glycan at days 30 and 90 post-infection, when compared to non-infected or even the initial time-points of infection (day 15 post-infection) (Figure 1B).

As compared to the *M. tuberculosis* strain H37Rv, the strain HN878 is known to be hypervirulent [35] and as we have recently described, C57BL/6 mice infected with low doses of H37Rv do not recapitulate features of active TB disease, showing instead a resistance phenotype [36]. Thus, we raised the hypothesis that the upregulation of epithelial SLeX might be associated with disease progression. In support of this hypothesis, we measured the expression of SLeX during infection in a different mouse strain, the susceptible C3HeB/FeJ mice [36,37]. Infection of these animals with the *M. tuberculosis* H37Rv strain, resulted in an increased expression of SLeX antigens in the lung, which was readily seen at day 30 post-infection (Figure 1C). Overall, we provide evidence for the up-regulation of SLeX antigens in the lung during experimental *M. tuberculosis* infection, in conditions associated with susceptibility.

### 3.2. M. tuberculosis Infection Up-Regulates the Transcription of Enzymes Controlling the Biosynthesis of SLeX on Core-2 O-Glycans

**Figure 2:**
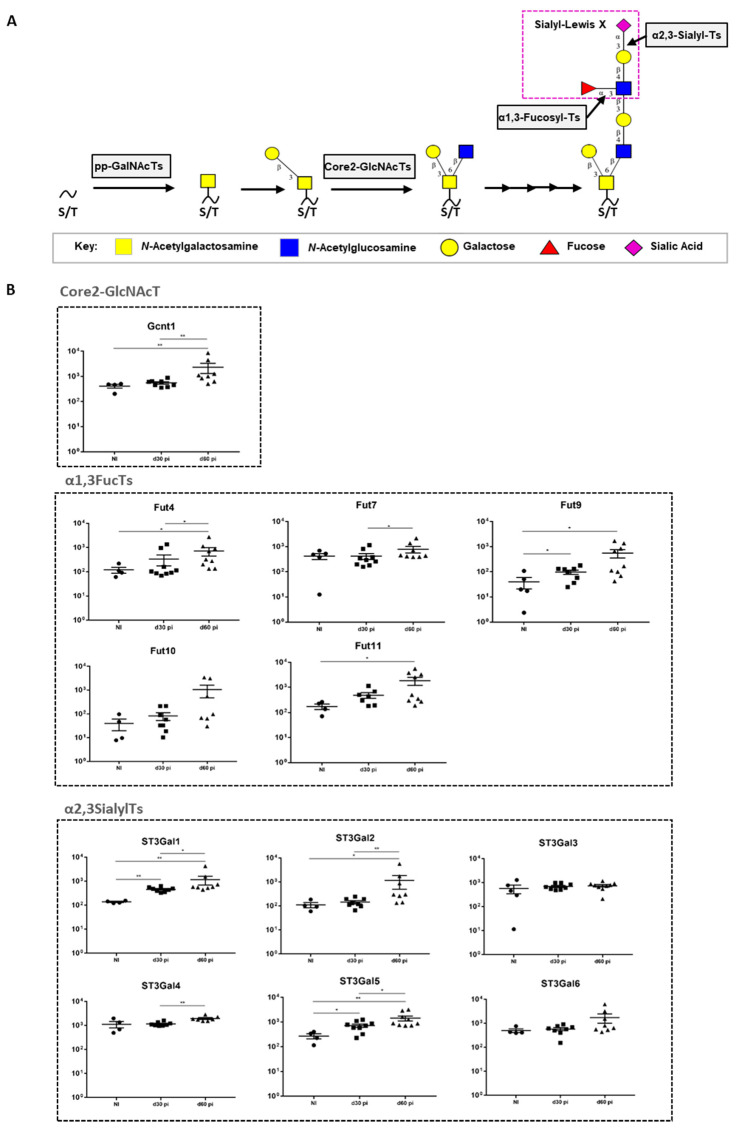
*M. tuberculosis* HN878 infection in C57BL/6 upregulates the transcription of several glycosyltransferase genes involved in the SLeX biosynthetic pathway. (**A**) Schematic representation of the biosynthetic pathway of SLeX-core 2 *O*-glycans. (**B**) The expression of the indicated glycosyltransferases, divided in 3 main categories (Core 2-GlcNAcT, α2,3SialylTs and α1,3FucosylTs), was measured by real-time PCR in lungs of C57BL/6 mice infected via aerosol with a low dose of *M. tuberculosis* strain HN878 on days 30 and 60 post-infection. Non-infected animals (NI) are shown as controls. Each dot represents one mouse of a total of 6–8 from 2 independent experiments. Statistical analysis was performed using one-way ANOVA with Tukey’s test for multiple comparisons. *, *p* < 0.05; **, *p* < 0.01. Monosaccharide symbols follow the Symbol Nomenclature for Glycans 38.

Taking into consideration that core-2 *O*-glycans are major carriers of terminal SLeX antigens and that their biosynthesis is regulated by the coordinated action of several glycosyltransferases [3,4,39], we measured, using real-time PCR, the transcript levels of key enzymes involved in the core-2 *O*-glycans SLeX antigens biosynthetic pathway (Figure 2A). Given our previous data (Figure 1), we did so in C57BL/6 mice infected with the *M. tuberculosis* strain HN878. Over the course of infection, we observed a significant upregulation on the transcription of several genes encoding enzymes participating in different steps of SLeX biosynthesis (Figure 2B). This was the case for the core 2 GlcNAcT Gcnt1, the α1,3-fucosyltransferases Fut4, 7, 9 and 11, and the α2,3-sialyltransferases St3Gal1, 2, 4 and 5 (Figure 2B). The transcriptomic analysis of glycosyltransferases on the H37Rv-infected C57BL/6 animals showed that, similar to what was observed on mice infected with HN878 strain, there was an increase on the expression of the α1,3-fucosyltransferases (Fut4, 9, 10 and 11), but no alterations were observed for the other enzymes evaluated (Figure S2). This increased expression in a more restricted number of enzymes could explain the lower amount of SLeX detected on the lung epithelium on these mice, when compared to the mice infected with a more virulent strain (*M. tuberculosis* HN878) (Figure 1). Our data reveals that the increased SLeX expression observed upon *M. tuberculosis* infection is accompanied by alterations at transcriptomic level, with up-regulation of enzymes that regulate the terminal SLeX decoration with fucose and sialic acid residues, constituting a specific glycosylation signature. Moreover, we demonstrate that the lung epithelium increase in SLeX is more evident for a hypervirulent strain of *M. tuberculosis*.

### 3.3. Lack of Gcnt1 Does not Preclude Increased Lung Sialylation upon M. tuberculosis Infection

**Figure 3:**
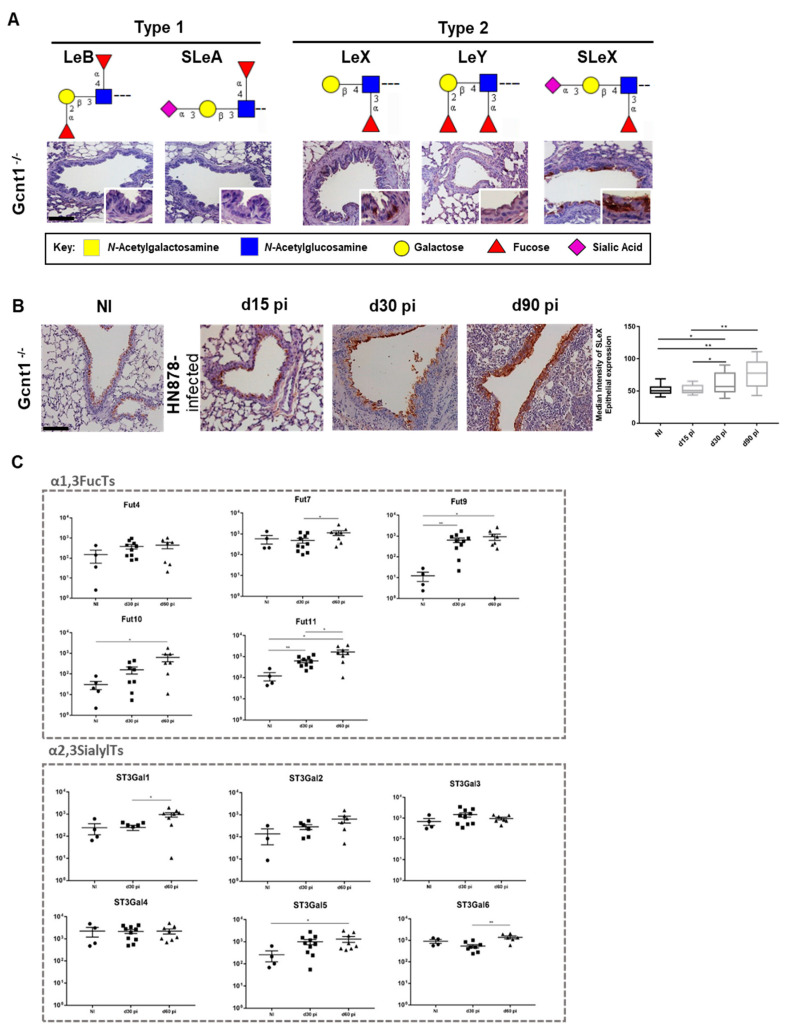
*Gcnt1* null mice infected with *M. tuberculosis* strain HN878 display lung increased SLeX expression concomitant with an up-regulated α2,3-SialylTs and α1,3-FucosylTs gene transcript signature. (**A**) Expression of type 1 (Lewis B (LeB) and Sialyl -Lewis A (SLeA)) and type 2 (Lewis X (LeX), Lewis Y (LeY) and Sialyl-Lewis X (SLeX)) Lewis antigens on *Gcnt1^-/-^* mice lung. Magnification 200× and inserts at 400×. (**B**) SLeX expression on lung epithelium of non-infected (NI) or *M. tuberculosis*-infected *Gcnt1^-/-^* mice. Mice were infected via aerosol with a low dose of *M. tuberculosis* strain HN878. Lungs were recovered at the indicated time-points post-infection, fixed and lung sections stained for SLeX. Magnification 200×. The panel on the right represents the quantification of epithelial expression of SLeX. (**C**) The expression of several glycosyltransferases involved on the SLeX biosynthetic pathways (α2,3SialylTs and α1,3FucosylTs) was measured by real-time PCR in lungs of *Gcnt1^-/-^* on days 30 and 60 post-infection. Non-infected animals (NI) are shown as controls. Each dot represents one mouse of a total of 6–8 from 2 independent experiments. Statistical analysis was performed using one-way ANOVA with Tukey’s test for multiple comparisons. *, *p* < 0.05; **, *p* < 0.01. Monosaccharide symbols follow the Symbol Nomenclature for Glycans [38].

We previously showed that mice lacking the GlcNAcT Gcnt1, a key enzyme for the biosynthesis of SLeX on core-2 *O*-glycans, display increased TB susceptibility and that both the hematopoietic and the stromal compartments contributed to such susceptibility [15]. That, together with our observation that *Gcnt1* transcription is upregulated upon infection, led us to question the impact of *Gcnt1* deficiency on the expression profile of Lewis antigens in the lung epithelium in naïve or *M. tuberculosis* infected mice. Naïve *Gcnt1^-/-^* animals displayed a pattern of Lewis antigens expression similar to WT C57BL/6, with absence of type 1 Lewis antigens and type 2 antigens detected on the bronchi and bronchiole epithelium (Figure 3A). Also similar to C57BL/6, the *Gcnt1^-/-^* showed a more pronounced expression of SLeX as compared to the other Lewis antigens tested (Figure 3A).

Infection of *Gcnt1*^-/-^ animals with *M. tuberculosis* strain HN878 resulted on increased SLeX expression on the lung epithelia during the course of infection, as demonstrated by the automatic quantification of staining, which was more pronounced at day 30 and 90 post-infection (Figure 3B). Similar to what was observed for the C57BL/6 animals, *M. tuberculosis* infection up-regulated the transcriptomic levels of several genes encoding enzymes involved on SLeX biosynthetic pathway on the *Gcnt1*^-/-^ infected animals (Figure 3C). Except for the Fut4 that was not significantly altered in the *Gcnt1* deficient animals, an up-regulation of the α1,3-fucosyltransferases, Fut7, 9, 10 and 11, was observed upon infection by *M. tuberculosis*, in line with the data obtained for C57BL/6 mice. Regarding the α2,3-sialyltransferases evaluated, we observed augmented transcripts of St3Gal1, 5 and 6 in *Gcnt1^-/-^* infected mice corroborating the overall increase in terminal α2,3-sialylation. These data suggest that the remodeling of the lung epithelia sialylated profile upon *M. tuberculosis* infection does not depend on a competent Gcnt1 enzyme.

## 4. Discussion

The role of glycans as chief players in defining pathogen tropism and colonization, as well as in numerous aspects of the immune response to infection, has been increasingly recognized over the last two decades [11,12,40,41]. However, the impact of the host glycosylation machinery, as well as its contribution to host susceptibility in TB is just starting to be unveiled [15,42,43]. Taking into consideration recent studies showing that deficiency in specific glycosyltransferases involved in Lewis antigens biosynthesis affect *M. tuberculosis* infection outcome [15,16,17], we evaluated how the epithelial expression of these terminal glycan epitopes in the lung was impacted by the infection. For this purpose, we started by analyzing the expression of Lewis antigens in the mouse lung epithelium and observed the exclusive detection of type 2 Lewis antigens and a main expression of the sialylated glycan SLeX. This observation is in accordance with previous reports showing predominance of type 2 Lewis structures and the glycosyltransferases involved in their biosynthesis in different mouse organs [18,27,44]. Furthermore, we observed that, similar to the augmented sialylation described to occur in response to infection by several other pathogens, *M. tuberculosis* infection also resulted in increased levels of SLeX. Interestingly, this increase in SLeX was mostly marked when mice where infected with the hypervirulent strain HN878, in comparison with the laboratory reference strain H37Rv. We have previously shown that SLeX is detected in lung sections of TB patients who were submitted to pulmonary surgery to treat TB [15]. Therefore, our data is in line with recent findings showing that mouse infection with the clinical isolate HN878 recapitulates more closely the pathogenesis of human TB disease, when compared with infection with the laboratory strain H37Rv [36]. In the same line, we found that C3HeB/FeJ mice that display increased susceptibility to *M. tuberculosis* infection presented marked levels of SLeX in the lung epithelium upon infection.

Cellular glycosylation is a complex process with multiple layers of regulation such as: glycosyltransferase gene transcription, availability of nucleotide sugar donors, relative amounts of enzymes competing for identical substrates, Golgi intracellular enzyme trafficking, and glycan turnover at the cell surface [5,45]. Our results reveal that the augment expression of SLeX in the mouse lung epithelium is accompanied by the transcriptional up-regulation of several glycosyltransferases involved in different enzymatic steps of SLeX biosynthesis. Supporting the differences that we observed regarding SLeX expression when comparing *M. tuberculosis* strains with different virulence features, experimental infection with *M. tuberculosis* H37Rv or HN878 also yield different glycosyltransferase transcriptomic signatures. Whereas infection with the HN878 led to significant lung up-regulation of several α2,3-sialyl- and α1,3-fucosyl-transferases, involved in the terminal decoration of SLeX, infection with H37Rv strain displayed fewer significant changes limited to the enzymes involved in the addition of the terminal fucose residues. Noteworthy, comparison of glycosyltransferases expression data in our infected mice with transcriptomic analysis of whole blood from active TB patients [15] reveals a common pattern, with identification of several homologous genes, therefore supporting a common mechanism across species. Furthermore, in line with the up-regulation of SLeX during the course of disease, a recent study showed that urinary levels of sialic acid, the terminal glycan of SLeX structure, discriminate patients with active TB from healthy controls and patients with non-tuberculous pulmonary diseases [46].

Although the molecular signaling pathways leading to up-regulation of glycosyltransferases in response to *M. tuberculosis* infection remain unknown, augmented epithelial sialylation has been previously associated with infection and inflammation [3,47]. A classic example is the up-regulation of terminal sialylated structures by *H. pylori*. These bacteria exploit the host glycosylation machinery to induce the expression of its adhesin’s glycan receptors promoting bacterial binding and a successful infection. Remarkably, the tissue expression levels of SLeX are also associated with the *H. pylori* strain pathogenicity, with more virulent strains promoting increased epithelial levels of this sialylated glycan [12,13]. In the case of *H. pylori* infection, it is acknowledged that both glycoproteins and glycolipids contribute for gastric glycophenotype switch to negatively charged glycans, such as SLeX. However, the carriers of SLeX in the lung epithelium in the context of *M. tuberculosis* infection remain elusive. Additionally, several cytokines, such as TNF and IL-1, have been previously shown to transcriptionally upregulate genes encoding glycosyltransferases [48,49].

Recently, we have shown that *Gcnt1* deficiency is associated with increased susceptibility to *M. tuberculosis* infection [15]. The Gcnt1 enzyme belongs to the core-2 GlcNAc transferases family, which are responsible for the initiation of the core-2 extension required for the generation of SLeX core-2 *O*-glycans. Therefore, we decided to explore firstly if *Gcnt1* abrogation would impact Lewis antigen expression in the lung epithelium, and secondly how lack of *Gcnt1* would affect the modulation of SLeX expression upon *M. tuberculosis* infection. We observed that *Gcnt1* deficiency did not change the Lewis antigens lung repertoire, and that *Gcnt1^-/-^* mice lung epithelium also displayed increased SLeX, supported by up-regulation of terminal α2,3-sialyl- and α1,3-fucosyl-transferases, in response to infection. Given the redundancy in the glycans biosynthetic pathways it is possible that the same enzymatic step may be catalyzed by different enzymes. In mice, two different enzymes Gcnt1 and Gcnt3 share core-2 GlcNAc-transferase activity [18]. To test the hypothesis of a *Gcnt3* compensatory activity in *Gcnt1* null mice, we evaluated the transcription levels of *Gcnt3*, upon infection but no significant changes were found when comparing WT and *Gcnt1^-/-^* mice (data not shown). Therefore, it is tempting to speculate that the overall increased sialylation of the lung may result from SLeX capping of different glycan carriers in C57BL/6 and *Gcnt1^-/-^* mice. From a biochemical perspective, it will be interesting to structurally analyze the glycan composition of the *Gcnt1^-/-^* lung to investigate for possible remodeling events. Of note, these remodeling events may involve *N*-glycans, as well as *O*-glycans, a hypothesis worth pursuing in the future.

Our findings reveal the modulation of SLeX during *M. tuberculosis* infection and highlight the significance of glycans and their biosynthetic pathways as possible new targets to modulate inflammation and immune response balance in TB.

## Figures and Tables

**Table 1:** Monoclonal antibodies used for Lewis antigens analysis by immunohistochemistry.

Antigen	Antibody Clone	Dilution (IHC)	Company/Reference
Lewis B (LeB)	BG-6 (T218)	1:50	Signet
Sialyl-Lewis A (SLeA)	CA19-9	1:500	Santa Cruz Biotechnology
Lewis X (LeX)	SH1	1:5	[29]
Lewis Y (LeY)	AH6	1:10	[30]
Sialyl-Lewis X (SLeX)	CSLEX-1	1:80	BD Biosciences

**Table 2:** Oligonucleotide sequences used for mice glycosyltransferases quantitative real-time PCR analysis.

Gene	Forward Primer	Reverse Primer
*Gcnt1*	5′- CGAAGGCCATGTTTCCAACGG -3′	5′- TCCGAAGACGCACACAGAGC -3′
*Fut4*	5′- ACCAGGAGGGAGCAGTGACG -3′	5′- TCCACACCCACCTCTGCCC -3′
*Fut7*	5´- GGACGACTTCAGCTCTGCCC -3′	5′- CGCCAAGCAAAGAAGCCACG -3′
*Fut9*	5′- TCCCATGCGGTCCTGATTCAC -3′	5′- TTCTGAAAGGGTGGCCTGGC -3′
*Fut10*	5′- GGGTGTGCAGGACATTAACC -3′	5′- AGCCTACTGTTTGCCCACAC -3′
*Fut11*	5′- GGGTGCTCAGTGTCTGTTCGG -3′	5′- CCCACGGCTCCTCCCTCTC -3′
*St3gal1*	5′- TCCTACAACTGCACAGCGTCG -3′	5′- TGTTTCGCCTGGTGCCTGG -3′
*St3gal2*	5′- GCTCTCTTCGGGTGTGGTTCC -3′	5′- ATGCTGTGGTGCGAGTAGGTG -3′
*St3gal3*	5′- CAGCAAGAAACCCAGACCAT -3′	5′- ATGAATGGCTCCGTCCATAG -3′
*St3gal4*	5′- GCTCCTGTGGCTGGCTACG -3′	5′- GGGTCAAAGTGGGCCGACTC -3′
*St3gal5*	5′- AGCCTCTTGGATATGCTGCCC -3′	5′- CGTTCCCAACAACCACACAGC -3′
*St3gal6*	5′- ATGGTGGCATTCCCGTAGTA -3′	5′- AAGTGCACCTCGCTGGTTT -3′
*Ubiquitin*	5′- TGGCTATTAATTATTCGGTCTGCA -3′	5′- GCAAGTGGCTAGAGTGCAGAGTAA -3′

## Data Availability

The data presented in this study is contained within this article or supplementary material.

## Supplementary Materials

The following are available online at https://www.mdpi.com/2076-2607/9/1/99/s1, Figure S1: Lung bacterial burdens obtained for the different experimental infections used in this study, Figure S2: Transcriptomic analysis of glycosyltransferase-encoding genes on C57BL/6 mice infected with H37Rv *M. tuberculosis* strain.
