# Supplementary material for: Mycobacterium tuberculosis Infection Up-Regulates Sialyl Lewis X Expression in the Lung Epithelium
Source: Microorganisms. 2021 Jan 4;9(1):99. doi: 10.3390/microorganisms9010099 (PMC7823657; doi:10.3390/microorganisms9010099)
Supplement: Supplementary file 1 [file microorganisms-09-00099-s001.pdf]

Supplementary Figure 1

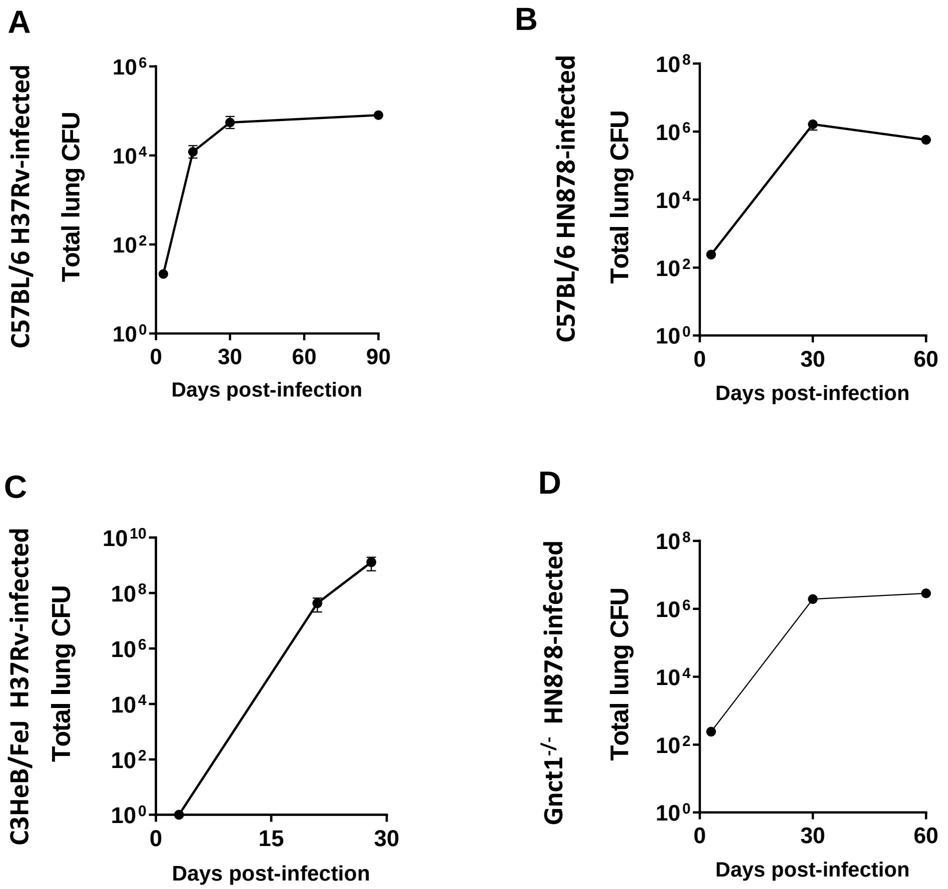

Supplementary Figure 1. Lung bacterial burdens obtained for the different experimental infections used in this study.

C57BL/6 mice were infected by aerosol with a low dose of *M. tuberculosis* strain H37Rv (**A**) or HN878 (**B**). C3HeB/FeJ mice were infected intravenously with H37Rv strain (**C**). *Gcnt1-/-* mice were infected by aerosol with a low dose of *M. tuberculosis* strain HN878 (**D**). At the indicated time-points post-infection, the lungs of infected mice were collected and the bacteria burden determined by CFU enumeration. Represented is the Mean±SEM for at least 5 mice per time point.

Supplementary Figure 2

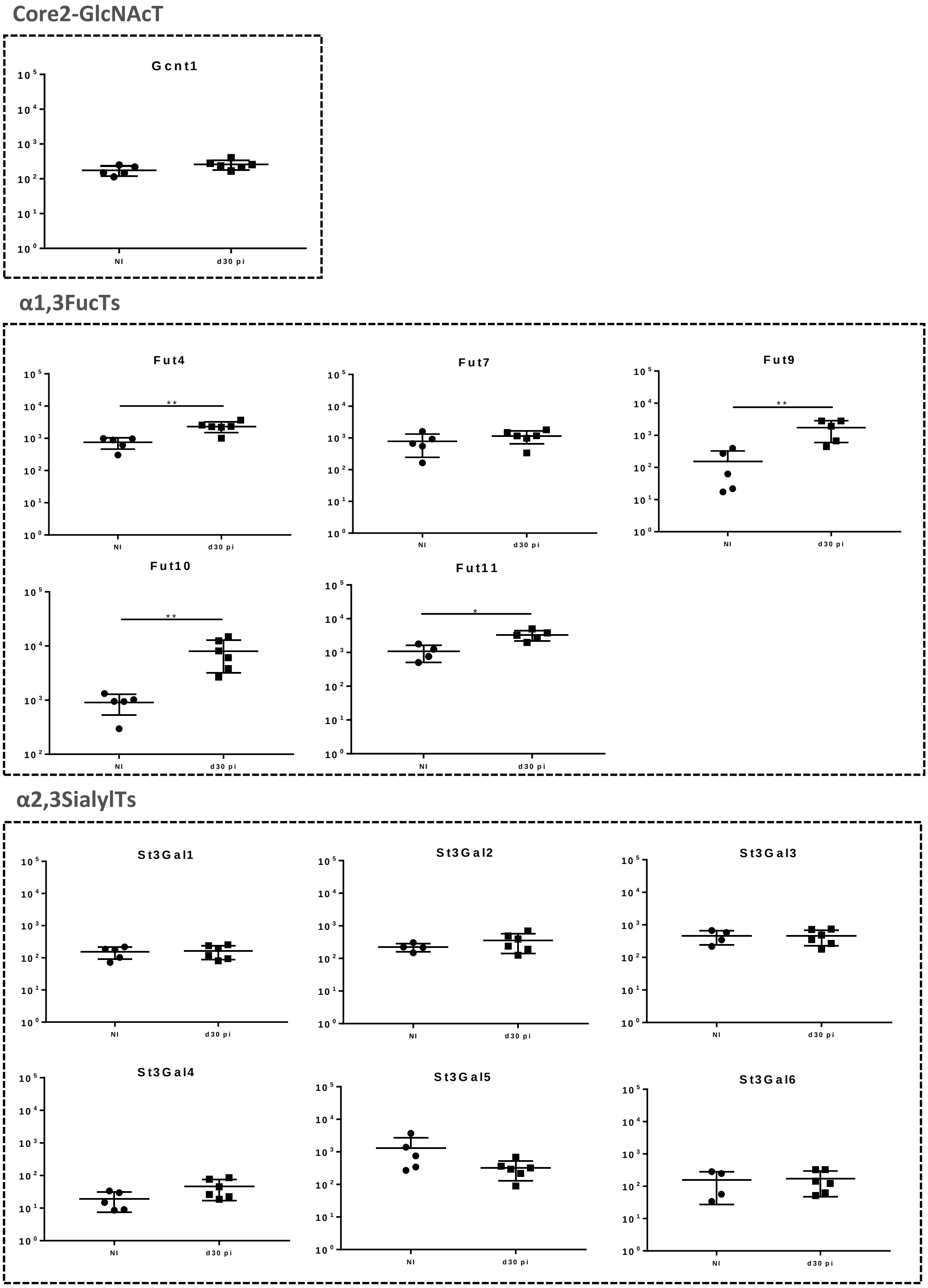

Supplementary Figure 2. Transcriptomic analysis of glycosyltransferase-encoding genes on C57BL/6 mice infected with H37Rv *M. tuberculosis* strain. The expression of several glycosyltransferases involved on the SLeX biosynthetic pathways (Core 2-GlcNAcT, α2,3SialylTs and α1,3FucosylTs) was measured by real-time PCR in lungs of C57BL/6 mice infected via aerosol with a low dose of *M. tuberculosis* strain H37Rv on days 15, 30 and 90 post-infection. Non-infected animals (NI) are shown as controls. Each dot represents one mouse of a total of 6-8 from 2 independent experiments. Statistical analysis was performed using one-way ANOVA with Tukey's test for multiple comparisons. *, $p < 0.05$; **, $p < 0.01$.
